# Take-Home Trial Comparing Fast Fourier Transformation-Based and Filter Bank-Based Cochlear Implant Speech Coding Strategies

**DOI:** 10.1155/2017/7915042

**Published:** 2017-09-13

**Authors:** Monique A. M. de Jong, Jeroen J. Briaire, Johan H. M. Frijns

**Affiliations:** ^1^ENT Department, Leiden University Medical Centre, Leiden, Netherlands; ^2^Leiden Institute for Brain and Cognition, Leiden University, Leiden, Netherlands

## Abstract

Previous studies have demonstrated no improved or deteriorated speech intelligibility with the HiResolution Fidelity 120™ speech coding strategy (HiResF120) over the original HiRes strategy. Improved spectral and deteriorated temporal sensitivities have been shown, making it plausible that the beneficial effect in the spectral domain was offset by the worsened temporal sensitivity. We hypothesize that the implementation of fast Fourier transform (FFT) processing, instead of the traditionally used bandpass filters, explains the reduction of temporal sensitivity. In this study, spectral ripple discrimination, temporal modulation detection, and speech intelligibility in noise were assessed in a two-week take-home trial with 3 speech coding strategies: one with conventional bandpass filters (HiRes), one with FFT-based filters (HiRes FFT), and one with FFT-based filters and current steering (HiRes Optima). One participant dropped out due to discomfort with both research programs. The 10 remaining participants performed equally well on all tasks with all three speech coding strategies, implying that FFT processing does not change the ability of CI recipients to discriminate spectral or temporal information or speech understanding.

## 1. Introduction

In an attempt to boost cochlear implant (CI) performance, the cochlear implant sound coding strategy “HiResolution Fidelity 120” (Advanced Bionics, Valencia, CA) (HiResF120) was developed [1]. This strategy implemented “current steering,” which facilitates stimulation of auditory nerve regions that are located in between physical electrode contacts. By simultaneously stimulating 2 adjacent electrode contacts with different weights, the peak of excitation shifts between the 2 contacts, creating an intermediate pitch percept [2–4]. Theoretically, this strategy generates up to 120 tonotopic positions, although psychophysical data reveal that most CI users are unable to discriminate such small differences in place pitch [4–7]. Although some studies reported improved speech understanding with HiResF120 [8–10], most were not able to demonstrate this [11–16]. Drennan et al. [17] compared HiResF120 with the traditional HiRes processing strategy and observed an improved spectral and a decreased temporal resolution, but no benefit for speech intelligibility in noise for HiResF120 users. Also, other studies reported an improved spectral resolution with the HiResF120 strategy [5, 6, 18], which could be attributed to the higher tonotopic precision of stimulation. As temporal cues are important for speech intelligibility in noisy environments [19–22], we hypothesize that the unchanged speech intelligibility in noise is because the beneficial effect in the spectral domain is offset by the reduced temporal sensitivity.

The cause of the detrimental effect on the temporal discrimination ability with HiResF120 is not known, but the way the frequency analysis is performed to enable current steering may be involved [17]. In the standard HiRes processing strategy, filter banks are implemented as 6th-order Butterworth bandpass filters in which spectral updating occurs at the pulse rate. To facilitate current steering, a filter bank based on fast Fourier transform (FFT) is used in HiResF120. These FFT filters provide a detailed spectral profile and are computationally efficient [23], making them of great interest in the implementation of speech processing designs. However, the 14.7 ms sliding window of these filters (256 pts Hamming Window) might cause temporal smearing, resulting in a decrease in temporal resolution. The present study examined the effect of FFT-based filter banks on temporal resolution, spectral resolution, and speech perception in noise.

## 2. Materials and Methods

### 2.1. Subjects

Eleven adults with postlingual deafness who had a HiRes90K device implanted with HiFocus1J or a CII HiFocus with positioner electrode array (Advanced Bionics, Valencia, CA) at the Leiden University Medical Centre (LUMC) participated in this study. All participants clinically used a Harmony processor with the HiRes speech coding strategy. The mean age was 60.6 (range: 43 to 74) years, the average duration of deafness was 26.4 (range: 4 to 67) years, and the average implant experience was 103 (range: 31 to 174) months. The mean phoneme score for open set Dutch monosyllabic (CVC) words during quiet conditions at 65 dB SPL (sound pressure level) was 89.6% (range: 76 to 96%) (Table 1). Subject 11 dropped out because of difficulty with the acceptance of the research speech processing strategies and due to a poor attention span.

### 2.2. Speech Coding Strategies and Programming

Participants were tested with 3 different speech coding strategies, all programmed on a Harmony processor. Strategy 1 (reference) was their standard clinical program, HiRes (Advanced Bionics, Valencia, CA), which is a bandpass filter-based strategy. More detailed information about this speech coding strategy is provided by Firszt [24]. The research strategies were HiRes FFT (strategy 2) and HiRes Optima (strategy 3). HiRes Optima is the current clinical standard strategy for Advanced Bionics implants, which is an energy efficient version of HiResF120. It saves energy by limiting current steering to only half of the area between 2 physical electrode contacts [25]. The distribution of current is expressed in alpha (*α*), where all current is delivered to the most apical electrode at *α* = 0 and to the basal electrode contact at *α* = 1. At *α* = 0.5, the current is equally distributed. HiResF120 applies current steering between *α* = 0 and *α* = 1, while HiRes Optima steers between *α* = 0.25 and *α* = 0.75. HiRes FFT (strategy 2) was identical to HiRes Optima, without the implementation of current steering, and it uses 16 instead of 15 channels for the FFT (see Table 2 for strategy characteristics).

The HiRes MAPs (MAP refers to programmed settings including T- and M-levels and stimulation rate, as well as other parameters) were transferred and adapted from the clinical software Soundwave to the research tool BEPS+ (Bionic Ear Program System+, Advanced Bionics, Valencia, CA), with which the 2 research strategies were programmed. Both strategies 2 and 3 were optimized by applying a preset gain profile, in which the signal is progressively attenuated with increasing electrode contact numbers (i.e., more basal electrode contacts). This gain profile results in a less sharp overall sound, thereby increasing the perceptual similarity with the clinical strategy. If the participant reported poor sound quality, individual MAPs were adjusted minimally, as is done in clinical practice. In Table 3, the fitting parameters for all subjects are shown. Three subjects (S3, S7, and S9) had up to four electrode contacts switched off in their HiRes MAP due to the clinical practice in our center at the time of hook-up. If this was the case, this pattern was copied to the HiRes FFT program. As it is impossible to copy this pattern to the HiRes Optima strategy and impedances on those electrodes were within normal ranges, the full electrode array was used for HiRes Optima fitting. Subject 1 had clinically switched off electrodes 3 and 4 because of relatively high impedances and electrodes 6 and 9 according to clinical practice. Only the high impedance electrodes were switched off for the research strategies. Subject S8 (bilaterally implanted) had clinically switched off electrode contacts 14–16 on the right side and 1–3 on the left side to compensate for interaural frequency mismatch caused by different intracochlear positions of the 2 electrode arrays. The same electrodes were used for the HiRes FFT and Optima strategies. Bilateral users (S7 and S8) were tested bilaterally.

### 2.3. Protocol

The participants were randomly assigned into 2 groups that participated in the study in a different order to avoid potential influence from auditory experience with the CI. To avoid outcomes due to learning effects rather than differences in strategy, the psychophysical test protocol was first completed with the HiRes strategy. These results were discarded here but used in a companion paper on learning effects. Subsequently, 2 weeks of at-home adjustment time was offered with one of the research strategies. When the subject returned, the test battery was repeated and the other research strategy was fitted on the processor. After another 2 weeks of practice at home, the second research strategy was evaluated. Final measurements with strategy 1 (the HiRes strategy) were obtained 2 weeks after finishing the trial.

### 2.4. Psychophysical Testing

All tests were conducted in a double-walled sound-attenuating booth. The sounds were presented at 65 dB SPL via a single loudspeaker, placed approximately 1 m from the listener at a straight angle that was in level with the listener's head.

A Flemish sentence test (LIST) was used to measure speech reception thresholds (SRTs) in speech shaped noise [26]. The standard LIST protocol was followed, but the level of the speech was held constant at 65 dB SPL to avoid loudness effects on speech discrimination. The noise level was adapted via a one-down, one-up procedure with step sizes of 2 dB, starting at 69 dB SPL. Five runs were obtained to determine the average SRT in dB signal-to-noise ratio (SNR).

To test spectral resolution, the Spectral-temporally Modulated Ripple Test (SMRT) as developed by Aronoff and Landsberger [27] was used. This one-up, one-down adaptive, 3-alternative, forced choice task determines the maximum number of ripples per octave (RPO), for example, the ripple density, which the listener can distinguish from 20 RPO. In the present study, the test was repeated 6 times to determine the average ripple density threshold.

Information about temporal sensitivity was obtained with a two-down, one-up adaptive forced choice task as adapted from Won et al. [28]. The modulation frequency of the amplitude-modulated wide band noise was 100 Hz, as this modulation frequency, when combined with ripple thresholds, accounts for the highest amount of speech variance [28]. Six tracking histories were conducted to determine the average modulation detection thresholds (MDTs) in dB relative to 100% modulation.

### 2.5. Subjective Assessment

To evaluate the subjective rating of speech coding strategies, the Speech, Spatial and Qualities of Hearing Scale (SSQ) was used [29]. The SSQ questionnaire is a measure for evaluating various aspects of hearing disability, of which the domains “quality of hearing” and “speech understanding” were assessed.

### 2.6. Statistical Analysis

A two-way repeated measures ANOVA with within-factors “strategy” (HiRes, HiRes FFT, and HiRes Optima) and “repetition number” (repetition number 1–5 or 1–6) was used to determine if there was a main effect of strategy, repetition number, and interaction between those 2 factors. SPSS Statistics Version 20 was used for calculations. A post hoc power analysis was conducted using the software package G^*∗*^Power [30]. The alpha level used for this analysis was *p* < 0.05 and the observed correlations among repeated measures were 0.8, 0.5, and 0.75 for the SMRT, MDT task, and LIST, respectively. Effect sizes *f* for the SMRT and MDT task were 0.28 and 0.58, based on data from the study of Drennan et al. (2010) [18]. For the speech-in-noise task, no effect was found by Drennan et al. (2010). Therefore, an effect size of 0.25, which is considered a moderate/clinically relevant effect, was chosen. The analysis revealed that the statistical power to detect the expected effect for the SMRT, MDT, and LIST results was 0.89, 0.96, and 0.80, respectively. From these results, we concluded that the statistical power with 10 subjects was sufficient.

## 3. Results

**Figure 1:**
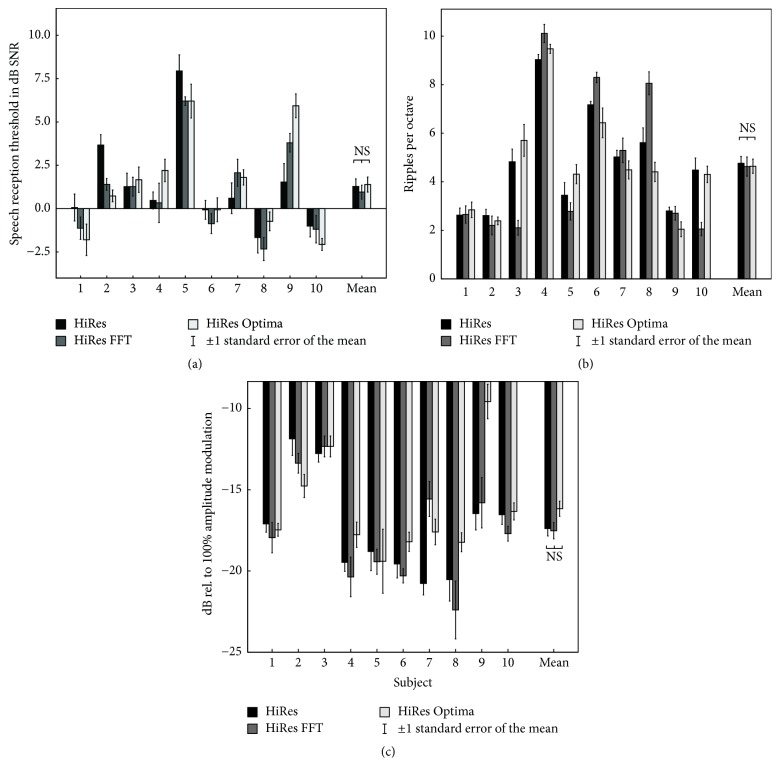
Individual and mean psychophysical results. Error bars indicate 1 SD. (a) Speech-in-noise intelligibility (LIST); (b) spectral ripple discrimination thresholds (SMRT); (c) amplitude modulation thresholds.

The results of the speech-in-noise test are shown in Figure 1(a). Mean SRTs were 1.3 dB SNR for HiRes, 0.96 dB SNR for HiRes FFT, and 1.4 dB SNR for HiRes Optima. The two-way repeated measures ANOVA failed to detect a statistically significant difference between speech coding strategies [Greenhouse-Geisser corrected *F*(1.23,11.1) = 0.396, *p* = 0.585]. Also, no significant effect of repetition number [*F*(4,36) = 2.2, *p* = 0.09] or interaction between strategy and repetition number [*F*(8,72) = 0.819, *p* = 0.589] was observed.

The individual and mean results of the spectral ripple test are shown in Figure 1(b). Mean SMRT scores were 4.76, 4.63, and 4.64 RPO for HiRes, HiRes FFT, and HiRes Optima, respectively. SMRT scores were not statistically significantly different across speech coding strategies [Greenhouse-Geisser corrected *F*(1.1,9.9) = 0.046, *p* = 0.86]. A significant effect of repetition number [*F*(45,5) = 2.862, *p* = 0.025] was found, whereas no interaction between strategy and repetition number [*F*(10,90) = 0.910, *p* = 0.527] was observed.

Individual and mean results of the MDT test are shown in Figure 1(c). The MDTs in dB relative to 100% modulation were −17.38, −17.52, and −16.17 dB for HiRes, HiRes FFT, and HiRes Optima, respectively. Although the results were numerically higher (worse performance) with HiRes Optima, there was no statistically significant effect of speech coding strategy, *F*(2,18) = 1.93, *p* = 0.175. No effect of repetition number [*F*(5,45) = 0.973, *p* = 0.445] or interaction between strategy and repetition number [*F*(10,90) = 1,519, *p* = 0.145] was found.

An additional paired *t*-test, comparing the average MDTs of HiRes FFT and HiRes Optima to final HiRes scores, was performed, but also this direct comparison between FFT and bandpass filter-based strategies could not demonstrate a significant effect (*p* = 0.403). Similarly, no significant effect of current steering on SMRT scores was found when comparing the average SMRT scores for HiRes and HiRes FFT to the HiRes Optima scores with a paired *t*-test (*p* = 0.882).

**Figure 2:**
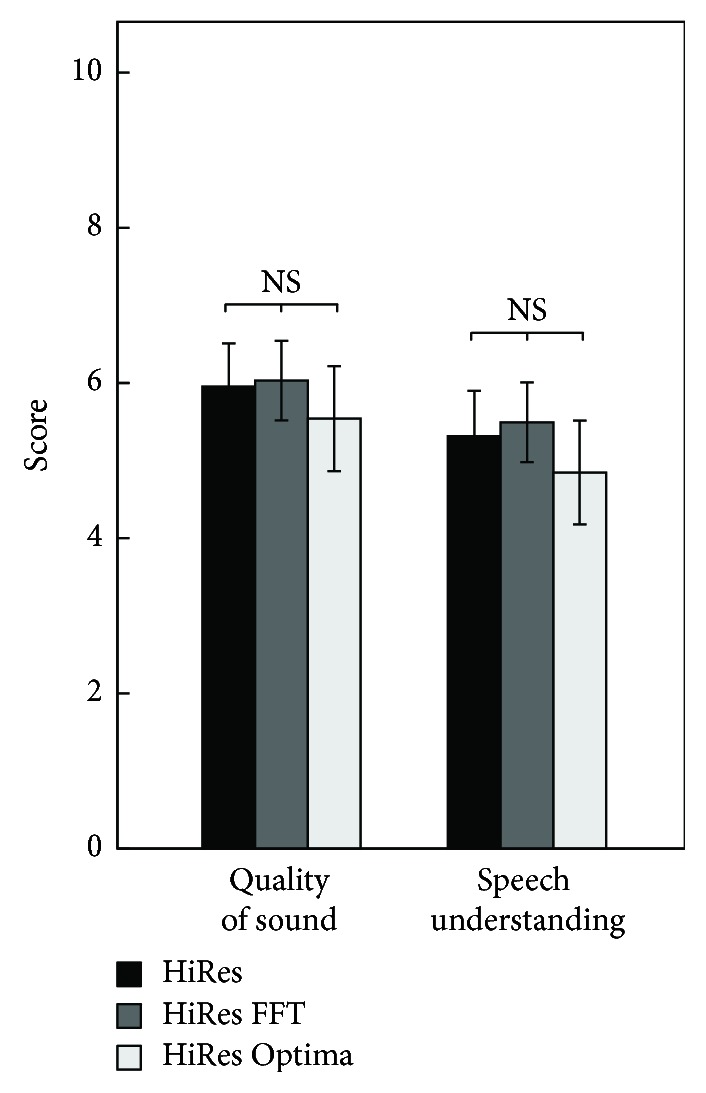
*Subjective rating* of processing strategies (SSQ) concerning quality of sound (left panel) and speech understanding in different listening conditions (right panel).

The means of the subjective ratings based on a 10-point scale are shown in Figure 2, separated in the quality of sound and speech understanding in different listening situations. On average, subjective quality of sound was rated 5.95, 6.03, and 5.54 with HiRes, HiRes FFT, and HiRes Optima [*F*(2,16) = 1,295, *p* = 0.3]. Speech understanding was rated as 5.32, 5.49, and 4.85, respectively [*F*(2,16) = 1.43, *p* = 0.268].

## 4. Discussion

This study evaluated 3 sound processing strategies, which used bandpass filters (HiRes), FFT filters (HiRes FFT), or FFT filters and current steering (HiRes Optima), to examine whether there is an effect of FFT processing. Speech intelligibility in noise was not statistically significantly different for the 3 speech coding strategies, implying that there was minimal influence from the combined changes to the type of filter bank, envelope extraction technique, or use of current steering. Considering the notion that prolonged experience with new strategies increases performance [15], one might argue that the optimal effect was not reached after 2 weeks of exposure to the strategies. Although no benefit has been seen with HiRes FFT and HiRes Optima, it is good to notice that also no acute detriment was observed when switching to these speech coding strategies. Moreover, many other research groups found no or only minor improvements on clinical abilities with HiResF120 as compared to HiRes [8, 17], which is in line with our results.

To study the sound processing strategies in more detail, more specific tests were needed. The SMRT and MDT tests are tests for spectral and temporal resolution, respectively. Both can be used in an acute setting and are correlated with speech recognition scores over time [28, 31–33]. No statistically significant benefit over standard HiRes was observed for spectral ripple discrimination with the HiRes Optima or HiRes FFT strategies, even while more electrode contacts were switched on with the HiRes Optima strategy in some subjects. This is in contrast with previous research, where improved spectral ripple discrimination was observed with HiResF120 [17, 18]. Also, Firszt et al. (2007) reported a decrease in just noticeable difference in pitch [5]. An explanation for our contradictory results might be that we used HiRes Optima, a more energy efficient version of HiResF120. Whereas HiResF120 applies current steering to the full area between 2 pairs of physical electrode contacts (between *α* = 0 and *α* = 1), HiRes Optima only steers current along a part of this area (between *α* = 0.25 and *α* = 0.75). This might explain the decrease in benefit in the spectral domain with HiRes Optima as compared to HiResF120, although no difference in speech understanding between these two strategies was found in a clinical study [25]. This could be explained by the fact that speech-in-noise tests are not sensitive enough to detect small differences between strategies and fine spectral detail may not be needed to achieve those levels of performance. To confirm the latter explanation, these 2 sound processing strategies (HiResF120 and HiRes Optima) should be investigated more extensively by comparing spectral ripple thresholds.

Although it seemed plausible that temporal smearing, caused by the wider time window of FFT processing, would lead to more difficulties in the temporal domain [17], our results do not confirm this hypothesis. Temporal modulation detection is not statistically significantly different between the speech coding strategies tested, although performance was numerically worse with HiRes Optima relative to HiRes FFT (*p* = 0.175).

Interestingly, this study showed a significant effect of repetition number within each SMRT test session, contrary to the companion study on learning effects. There, only a borderline significant effect (*p* = 0.052) was observed when comparing the first and last measurements in a sequence of six. However, in that paper, comparison of baseline and 6-week SMRT and TMTF scores revealed a clear learning effect over time. Therefore, baseline HiRes scores were discarded in the present study, and only final HiRes scores were used as a reference for HiRes FFT and HiRes Optima. Nevertheless, it turned out that even if baseline HiRes scores would have been used, no significant effect of speech coding strategy on both SMRT (*p* = 0.071) and MDT (*p* = 0.126) scores could be demonstrated.

## 5. Conclusion

The present study compared the influence on several aspects of CI performance of FFT-based filter banks and the traditional bandpass filters as used in the HiRes speech processing strategy. Neither detrimental nor beneficial effects were found in spectral and temporal resolution, or speech intelligibility in noise. The known benefits of FFT filters, for example, their computational efficiency, encourage their implementation in future speech coding strategies.

## Figures and Tables

**Table 1:** Subject characteristics.

Subject	Gender	Age (yrs)	Etiology	Deafness (yrs)	CI side	CI experience (mos)	CVC (Ph%)	Implant
S1	Male	60	Familial	46	Left	160	93	Clarion CII
S2	Male	51	Progressive	25	Right	124	76	HiRes 90K
S3	Female	74	Familial	22	Left	114	93	HiRes 90K
S4	Male	58	Unknown	11	Right	31	93	HiRes 90K
S5	Male	67	Familial	67	Right	96	86	HiRes 90K
S6	Male	57	Familial	45	Right	174	96	Clarion CII
S7	Female	67	Meningitis	6	Left + right	73	88	HiRes 90K
S8	Female	43	Meningitis	5	Left + right	59	95	HiRes 90K
S9	Female	66	Unknown	35	Right	105	84	HiRes 90K
S10	Female	59	Type II Usher	4	Right	46	89	HiRes 90K
S11	Female	65	Meniere	25	Right	149	92	Clarion CII

CI: cochlear implant; CVC: Dutch phonetically balanced monosyllabic consonant-vowel-consonant words; Ph%: percentage phonemes correct.

**Table 2:** Speech coding strategy characteristics.

Strategy	Filter bank	Envelope extraction	Stimulation mode	Range of alpha	# spectral channels	Spacing of filters
HiResolution	Butterworth	HWR + LPF	Monopolar	—	16	Logarithmic
HiResolution FFT	FFT-based filters	Hilbert envelope	Monopolar	0	16	Logarithmic
HiResolution Optima	FFT-based filters	Hilbert envelope	Dual-electrode	0.25–0.75	135	Logarithmic

FFT: fast Fourier transform; HWR: half wave rectifier; LPF: low-pass filter.

**Table 3:** Fitting parameters.

Subject	HiRes			HiRes FFT			HiRes Optima		
	# channels	Pulse width	Pulse rate (PPS)	# channels	Pulse width	Pulse rate (PPS)	# channels	Pulse width	Pulse rate (PPS)
S1	12	21.6	1547	12	10.8	1547	12	25.1	1657
S2	16	21.6	1450	16	21.6	1450	15	18.0	1856
S3	12	21.6	1933	12	21.6	1933	15	26.0	1280
S4	16	21.6	994	16	31.4	994	15	43.1	773
S5	16	21.6	1450	16	21.6	1450	15	19.8	1687
S6	16	21.6	1450	16	21.6	1450	15	23.3	1428
S7	12/12	43.1/21.6	967/1933	12/12	43.1/21.6	967/1933	15/15	49.4/21.6	675/1856
S8	13/13	21.6/21.6	1785/1785	13/13	21.6/21.6	1785/1785	12/12	23.3/26.0	1785/1477
S9	12	10.8	1547	12	10.8	1547	15	29.6	750
S10	16	21.6	1450	16	21.6	1450	15	23.3	1428
S11	8	21.6	1450	—	—	—	—	—	—

PPS: pulses per second.
